# Intellectual disability rights and inclusive citizenship in South Africa: What can a scoping review tell us?

**DOI:** 10.4102/ajod.v7i0.396

**Published:** 2018-04-25

**Authors:** Charlotte Capri, Lameze Abrahams, Judith McKenzie, Ockert Coetzee, Siyabulela Mkabile, Manuel Saptouw, Andrew Hooper, Peter Smith, Colleen Adnams, Leslie Swartz

**Affiliations:** 1 Department of Psychiatry and Mental Health, University of Cape Town, South Africa; 2 Alexandra Hospital, Western Cape Government, South Africa; 3 Lentegeur Psychiatric Hospital, Department of Health, South Africa; 4 Department of Health & Rehabilitation Sciences, University Of Cape Town, South Africa; 5 Department of Psychology, Stellenbosch University, South Africa

## Abstract

**Background:**

Intellectual disability (ID) is the most prevalent disability in the world. People with intellectual disability (PWID) frequently experience extreme violations of numerous human rights. Despite greater prevalence in South Africa than in high-income countries, most ID research currently comes from the Global North. This leaves us with few contextually sensitive studies to draw from to advance inclusive citizenship.

**Objectives:**

Our scoping review aims to investigate pertinent ID rights issues in South Africa, synthesise quantitative and qualitative studies, and provide a synopsis of available evidence on which to base future work. We aim to clarify key concepts, address gaps in the literature and identify opportunities for further research.

**Method:**

We followed strict eligibility criteria. Medical subject heading terms were entered into seven databases. Seven reviewers worked independently, two per paper. Quantitative and qualitative data extraction forms were designed. We followed Preferred Reporting Items for Systematic Review and Meta-Analysis (PRISMA) guidelines and registered a protocol. An inductive approach enabled a thematic analysis of selected studies.

**Results:**

By following PRISMA guidelines, 82 studies were assessed for eligibility of which 59 were included. Ten sub-themes were integrated into four main themes: the right not to be discriminated against, the right to psychological and bodily integrity, the right to accommodating services and challenges to rights implementation.

**Conclusion:**

People with intellectual disability face compound difficulties when trying to assert their constitutionally entitled rights. This ongoing project requires serious commitment and action. Statutory obligations to nurture every South African’s human rights naturally extend to PWID and their supporters who forge ahead in a disabling environment.

## Background

Most people with disabilities live in low-income countries and represent nearly a quarter of the world’s poorest people (Emerson 2007; Groce et al. 2011a, 2011b). Against this backdrop, approximately 200 million people live with intellectual disability (ID), making it the world’s most prevalent disability (World Health Organization [WHO] & World Bank 2011).

**FIGURE 1:**
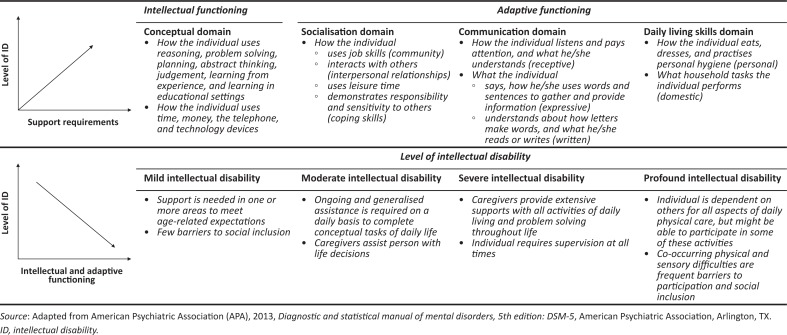
Continuum of support needs according to severity of intellectual disability.

Medical definitions ascribe ID to deficits in intellectual and adaptive functioning across various domains with onset during the developmental period (AAIDD 2017; American Psychiatric Association [APA] 2017; Crnic et al. 2017). The American Psychiatric Association no longer categorises ID by quantified performances on intellectual ability assessments (APA 2013). Individuals now shift along a continuum of domains that include conceptual, social and practical functioning, and that inform on mild, moderate, severe or profound ID. Supported decision-making and individualised care can help people with intellectual disability (PWID) negotiate limitations across compromised domains. Limitations are unlikely to be reversible, but adaptive skills can be developed in suitable environments. This will require types of assistance that vary according to levels of support needs and severity of ID (Figure 1).

Regardless of subjective experiences, the medical model regards impairment as an inherent problem of the individual that is addressed through medico-psychological skill (Nash & Navias 1992; Mckenzie & Macleod 2012a; Roy, Roy & Roy 2012). In South Africa, there are particular dedicated ID treatment sites, and special education is provided in separate schools on medico-psychological recommendation (Engelbrecht, Oswald & Forlin 2006; Mckenzie & Macleod 2012a).

The social model of disability resists pathologising impairment by separating it from disability (Goodley 2001; Shakespeare 2017; Swartz et al. 2012). Individuals may live with various impairments, but their political and social environments do the disabling. The impairment alone ‘is not sufficient for disablement to occur. What disables people – what *makes* people disabled – is how society responds to the impairments’ (Swartz 2010:27–28, own italics). Rather than locating disability in the person, it is viewed as the result of interactions among disabling physical environments and unequal social relationships that further encumber impairment (Young & Berry 2016). It separates bodily impairment from a socio-political unwillingness to accommodate an individual’s needs (Mckenzie & Macleod 2012a; Young & Berry 2016). The social model of disability does not escape criticism either. Disability might be a function of physical and socio-political environments, but competence can be interactively achieved among individuals and enabling environments (Mckenzie & MaCleod 2012b; Pillay 2003). In this way, individuals are enabled and environments become impairment competent. Moreover, some disabled people are proud of bodies marked by prejudice and chipped away by the norms of a predominantly able-bodied world (Hughes et al. 2017).

By synthesising the medical and social models, the WHO (2002) offers an integrative bio-psycho-social model of disability. The International Classification of Functioning, Disability and Health (ICF) frames biological, individual and social aspects of health as inseparable. Disability as a consequence of disease or socio-political ignorance shifts to a site of interacting health, environmental and personal factors (Mji et al. 2017). At any given time and in any combination, these factors can facilitate or hamper a person’s functioning (Selb 2017).

Finally, if we appreciate disability as fluid and situational, we are no longer polarised or opposed as enabled or disabled, but occupy different spaces on the continuum of ‘changing states of impairment and health’ (Swartz et al. 2012:1). By drawing on one another’s experience of disability and able-bodiedness, it becomes possible for us to shift and meet along this continuum and reach a ‘richer understanding’ of living with ID (Swartz et al. 2012:8).

The issue of societal inclusion and exclusion of PWID on psycho-medical, socio-political or accessibility grounds touches on core questions of rights claims, justice, citizenship, equality, resources and protections. People with intellectual disability may have limitations in advocating for themselves and ensuring that their best interests are prioritised. Not affording PWID support or opportunity to contribute to policies that directly impact their lives can be viewed as morally abusive (Kittay 2009:620). Disenfranchising PWID maintains vulnerability to exploitation, marginalisation and inequality in systems where the distribution of care and services hardly occurs in their favour and very seldom adequately (Adnams 2010). Without appropriate supportive decision-making or opportunity to assert access to recourse with assistance, PWID are subjugated to whatever form care may take and at risk of abuse and neglect (Makgoba 2016).

Driven by misconception, fear and lack of knowledge, discrimination against PWID can result in isolation, segregation, stigma and death (Byrne 2017; Flores 2017; Lindau et al. 2017; Maclean et al. 2017; Makgoba 2016; Thornicroft et al. 2007; Wissink et al. 2017). Only 2 out of every 100 children with disabilities receive schooling in developing countries (Cramm et al. 2013; Du Plessis 2013). Compared to the general population and other disability groups, PWID are more often unemployed or underemployed because of low expectations of competence (‘they’ can’t do much) and high expectations of problems (‘they’ are difficult to work with) (Carvalho-Freitas & Stathi 2017; Merrells, Buchanana & Waters 2017; WHO 2011; Wilson et al. 2017).

### A South African setting

South Africa exceeds ID prevalence in high-income countries (Maulik et al. 2011; McKenzie 2016; Tomlinson et al. 2014). To this point, foetal alcohol spectrum disorder (FASD) is an easily preventable cause of ID, but South Africa’s prevalence rate of 6% – 9% is one of the world’s highest (Adnams 2010; De Vries et al. 2013; Roozen et al. 2016; Urban et al. 2008).

Accurate South African data on ID prevalence among 2–9-year-old children were last gathered in the 1990s (Christianson et al. 2002; Kromberg et al. 1997, 2008). During the 2011 national census, ID was not directly measured, and statistics on children with disabilities aged 0–4 years were not profiled (Statistics South Africa [SSA] 2014). Current accurate South African ID data are rare (Du Plessis 2013; Fujiura, Rutkowski-Kmitta & Owen 2010). At last count, 3.2% of people aged 5 years and older have mild and 1% of people have severe difficulties ‘in remembering or concentrating’ (SSA 2014:34).^1^

Despite ratifying the United Nations Convention on the Rights of Persons with Disabilities (UNCRPD) (The United Nations [UN] 2006) a decade ago, South Africa’s dualist legal system has yet to assimilate the international conventions into domestic legislation on behalf of PWID (Huus et al. 2015). Lawmakers’ lack of understanding of what ‘intellectual disabilities actually *mean*’ was even officially gazetted in parliament (Department of Social Development [DSD] 2015, own italics). Additionally, although the White Paper on the Rights of Persons with Disabilities (DSD 2016) commits to PWID, it makes little mention of how these commitments will be implemented or monitored (De Vries et al. 2013; Drew et al. 2011; Kopel 2017; Officer & Shakespeare 2013; Roy et al. 2012).

Because of shortage of educational programmes for children with intellectual disability (CWID), parents in countries like South Africa easily become unpaid caregivers because care burdens and lack of support hinder their pursuit of employment (Geiger 2012; Mckenzie & McConkey 2016). Being out of school also limits exposure of CWID to formal teaching on sexual health programmes (Rohleder & Swartz 2009; WHO 2011). This is problematic, because more than two-thirds of adolescents with ID are at risk of sexual abuse before they turn 18, whereas up to 83% of women and 32% of men with ID are at increased risk of being sexually assaulted in their lifetime (Byrne 2017; Peckham 2007).

Other frequently violated rights of PWID in South Africa pertain to physical abuse, exclusion, barriers to accessing medical and mental health services, involuntary confinement, denial of marriage or parenting, financial exploitation, unemployment, occupational restrictions and living safely outside of institutions (Drew et al. 2011; Erasmus, Bornman & Dada 2016). These rights are violated in public, family homes, places of education and work, care centres, health care settings, police stations, courts and civic offices (Drew et al. 2011).

As yet, we know little about South African evidence-based studies on which to build better ID rights practice. Most ID research comes from high-income countries where PWID enjoy various government and community supports but is not always applicable to settings in which most PWID live (Glicksman et al. 2017; Groce et al. 2011a, 2011b; McKenzie, McConkey & Adnams 2013a; Robertson et al. 2012). For this reason, we aim to investigate pertinent South African ID rights issues, clarify key concepts, synthesise quantitative and qualitative studies and provide a synopsis of existing evidence (Arksey & O’Malley 2005; Daudt, Van Mossel & Scott 2013; Harden 2010; Peters et al. 2015).

## Aim

The primary aim of our scoping review was to collect all literature published in peer-reviewed journals on ID rights in South Africa over the past 25 years (1992–2017). We set out to study the ID advocacy, awareness and rights promotion research; describe outcomes of studies on realising human rights entitlements; and identify publications that address claims to citizenship of South African PWID. Following Peters et al.’s (2015) suggestions for scoping reviews, our objectives were guided by questions specific to this study:

- What do we know about the state of human rights of PWID in South Africa?
- Are there barriers jeopardising rights realisation?
- Are PWID participating in socio-political lives of communities?
- Is rights advocacy needed?
- Which studies can we use to address these questions?

We also aim to identify areas for future investigation by highlighting gaps in the available research.

## Method

We followed the Preferred Reporting Items for Systematic Review and Meta-Analysis (PRISMA) guidelines and registered a protocol with PROSPERO (CRD42016036100). An inductive approach accommodated thematic analyses of selected studies.

### Eligibility

Included studies are on rights of PWID in South Africa published between 1992 and 2017. Research sites and participants are in South Africa. Comparative country studies on ID had to include South Africa (World Bank 2017). Eligibility criteria for inclusion in this review are summarised in Table 1.

**TABLE 1:** Eligibility criteria for inclusion of studies in this scoping review.

Inclusion criteria	Exclusion criteria
Children and adults with ID	Studies on disability but not ID Studies on co-occurring presentations in which ID is not necessarily included by definition, or syndromes in which ID is a variable outcome
Human rights	Human rights study is not on rights of PWID
Research sites and participants are in South Africa	Study is not on South Africa
Peer-reviewed academic journal articles	Study not in blind peer-reviewed journal (e.g. in a predatory journal)
Published between 1992 and 2017	Studies outside time span
Full text available	Full-text unavailable (please see ‘Information sources’)
English	Languages other than English
Studies on ID in other unequal societies had to include South Africa (World Bank 2017)	Studies on ID in Africa and southern Africa but not South Africa Irrelevant database returns (e.g. South Carolina)

ID, intellectual disability; PWID, people with intellectual disability.

Reviewer panel consensus minimised study selection bias by resolving issues like the eligibility of studies on FASD and autism spectrum disorder (ASD). Intellectual disability is not required for an FASD diagnosis, and low or average intellect is actually more common in FASD (Royal College of Psychiatrists [RCP] 2001). Studies with participants with FASD and low or average intellect were thus excluded from this review (RCP 2001).

Although developmental disorders and ID coexist and share associations, ASD may not have caused ID and not all individuals with ASD have ID (RCP 2001). Conversely, studies that included, for example, people with Down syndrome (DS) and phenotypical ID were eligible for review (RCP 2001).

### Information sources

We searched seven databases (Web of Science, PubMed, Scopus, ERIC, Africa-Wide Information/NiPAD, African Journal Archive and African Index Medicus) to increase the likelihood of locating South African publications among international studies. Electronically unavailable records were hand searched, authors were contacted or subject librarians at Stellenbosch University were approached. Backward and forward citation searches identified additional studies. The last search ran on 31 August 2017 which was also the author contact cut-off date.

### Search strategy

Data were sought for outcome, setting and participants by using medical subject headings (MeSH) terms (Box 1) as per systematic scoping review practice.^2^ Searches were sensitised for time span (1992–2017) and English as publication language. English is a compulsory subject in South African schools and the language of business and government. It is also the preferred instruction medium in most tertiary institutions from which ID practitioners and researchers graduate (Dictionary Unit for South African English 2016; Donohue, Bornman & Granlund 2015).

Box 1 Search by medical subject heading terms.

- ***TOPIC:*** ‘Intellectual disability’ OR ‘developmental disabilities’
- ***AND* TOPIC:** ‘South Africa’ *(includes Republic of South Africa)*
- ***AND* TOPIC:** ‘Adult’*(includes aged, middle aged, young adult, aged 80 and over, frail elderly)* **OR** ‘adolescent’ **OR** ‘child’ *(includes child, preschool)* **OR** ‘infant’ *(includes newborn)*
- ***AND* TOPIC:** ‘Human right’ (*includes rights, human)*

### Study selection

In an attempt to minimise risk of bias across studies that may affect the overall review results, seven reviewers worked independently, two per paper, to assess each study according to the review’s eligibility criteria (C.C., O.C., J.M., L.A., M.S., S.M., A.H.). Three experts were available for data verification (P.S., C.A., L.S.). Future systematic reviewers can critically appraise individual bias within studies by means of checklists suitable to study design and available in the public domain as offered in Table 2. Individual studies can be assessed for evidence of bias reduction by purposeful design or by ways in which authors acknowledge any bias that might affect individual study results.

**TABLE 2:** Critical appraisal checklists.

Action research studies	Action research designs (Greenhalgh et al. 2008:242–244)
Before-and-after studies Can be (non) randomised and/or (non) controlled	Before-and-after study designs (HEBW)
Case control studies	Case control study (CASP)
Cohort studies	Cohort study (CASP)
Cross-sectional studies	Cross-sectional study (CASP)
Diagnostic studies	Diagnostic study (CASP)
Mixed-method case studies	Mixed-methodology case study (Greenhalgh et al. 2008:239)
Opinion and analysis	Narrative, expert opinion and text (JBI)
Prevalence studies	Studies reporting prevalence data (JBI)
Qualitative case studies	Qualitative case study (Atkins & Sampson 2002)
Qualitative studies	Qualitative studies (SURE)
Quantitative case studies	Quantitative case study (CEBMa)
Randomised controlled trials	Randomised-controlled-trials (SURE)
Reviews	Review studies (PRISMA)
Single participant case designs Medical case reports	Single participant case designs (PsycBITE)

### Data collection

Data extraction forms for quantitative and qualitative studies used PICOS (Population, Intervention, Comparison, Outcome, Study design) and SPIDER (Sample, Phenomenon of Interest, Design, Evaluation, Research type) elements, respectively (Cooke, Smith & Booth 2012) (Tables 3a and 3b).

**TABLE 3a:** Examples of data extraction forms – Population, Intervention, Comparison, Outcome, Study design: For quantitative studies.

First author	Population, study size and setting	Intervention, comparison, and/or effect size	Outcome	Study design
Ali (2015)	*n* = 191 Cape Town	Perceived stigma of intellectual disability tool (South African version). Significant interaction between ethnic group and level of intellectual disability (*F* = 3.74, *p* = 0.01). Lower age significantly associated with stigma (*p* = 0.02).	Younger people with intellectual disability and those with mild intellectual disability from black ethnic communities experience more stigma.	Case study (Quantitative)
Saloojee (2007)	*n* = 156 Orange Farm, Soweto, Gauteng	Screening questions, clinical observation, semi-structured interviews. Children with motor impairments were more likely to receive rehabilitation than those with intellectual impairment (*p* < 0.0001).	Little evidence found of cooperation between the health, education and social development departments.	Case study (Quantitative)

**TABLE 3b:** Examples of data extraction forms – Sample, Phenomenon of Interest, Design, Evaluation, Research: For qualitative studies.

First author	Sample and setting	Phenomenon of interest	Design	Evaluation	Research type
Engelbrecht (2003)	*n* = 55 Gauteng and the Western Cape	Inclusion (education services)	Questionnaire, in-depth structured interviews	Including learners with intellectual disability in mainstream classes is stressful for teachers.	Case study (Mixed method)
McKenzie (2012a)	*n* = 85 East London, Eastern Cape	Rights discourses	Q-methodology	The three rights claims (participation, special services and protection) should be re-examined through the lens of an ethics of care that enables participation, and that reconsiders reciprocity and interdependence.	Grounded theory study

### Ethical considerations

Although this systematic review did not require ethical clearance, it is based on literature sought for a research study that obtained ethical clearance from the Health Research Ethics Committee of Stellenbosch University’s Faculty of Health Sciences (Federal Wide Assurance Number: 00001372, Institutional Review Board Number: IRB0005239).

## Results

**FIGURE 2:**
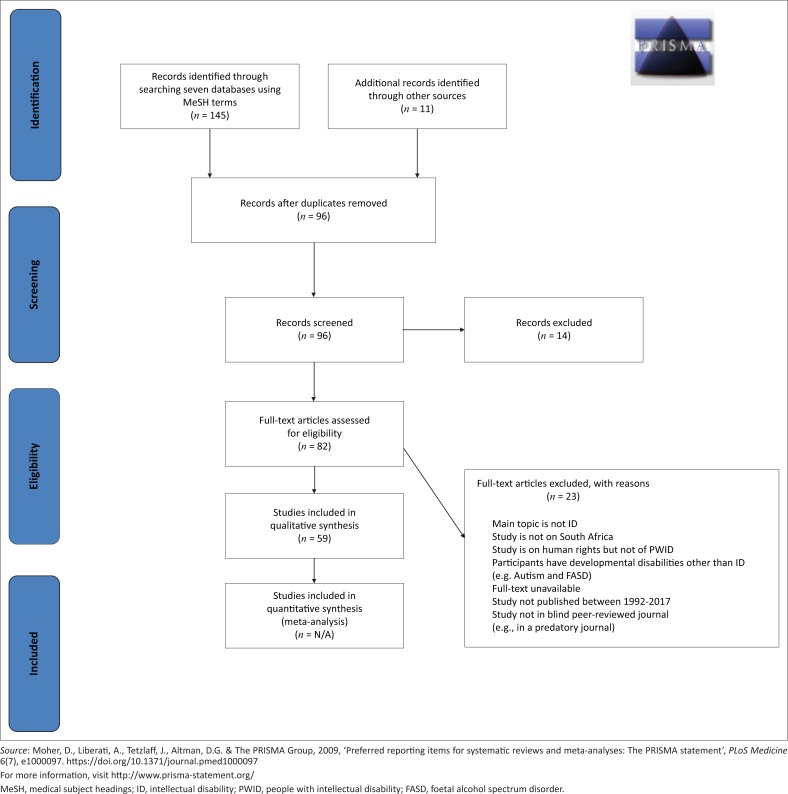
Preferred reporting items for systematic review and meta-analysis flow diagram – Results of study selection.

By following PRISMA guidelines, 156 records were identified overall. Ninety-six studies remained after deleting duplicates. Two reviewers screened each abstract resulting in 14 exclusions. Eighty-two studies were assessed for eligibility of which 59 were included for qualitative synthesis (Figure 2).

Characteristics of included papers (*n* = 59) are summarised in Table 4 in reference to alphabetically ordered first author, topic, study design and outcome. They are discussed in more detail below. Studies made use of quantitative (*n* = 26), qualitative (*n* = 31) and mixed-methods (*n* = 2) designs.

**TABLE 4:** Characteristics of included studies (*n* = 59).

	First author	Topic	Study design	Outcome
1.	Ali (2015)	Right not to be discriminated against	Case study (Quantitative)	Younger people with ID and those with mild ID from black ethnic communities experience more stigma.
2.	Bornman (2016)	Protection against abuse	Multi-method study (Qualitative)	Development of social stories to use in a sexuality and relationship training programme for women with ID.
3.	Bornman (2011)	The right to be understood	Action research (Qualitative)	Development of communication boards.
4.	Bornman (2002)	The right to be understood	Case study (Mixed method)	Nurses’ knowledge and skills in dealing with ID, PWID and caregivers is inadequate. Need for skills training identified.
5.	Bornman (1996)	The right to be understood	Case study (Quantitative)	38% prevalence rate of non-speaking CWID.
6.	Calitz (2011)	Bodily and psychological integrity	Opinion and analysis (Qualitative)	Need future research on best way to assist PWID to navigate psycho-legal settings.
7.	Calitz (2014)	Protection against abuse	Case study (Quantitative)	Burden of evidence to secure convictions rests on investigations and physical evidence.
8.	Calitz (2007)	The right to competence and capacity	Case study (Quantitative)	It is possible for a person with ID to be triable and accountable.
9.	Capri (2012)	Access to services	Opinion and analysis (Qualitative)	Excluding PWID from participating in research can be a human rights violation.
10.	Dada (2013)	Communicative inclusion	Case study (Quantitative)	Symbols used were relatively iconic to participants. Iconicity may be enhanced by modifying them according to age, culture and language.
11.	Dickman (2005)	Bodily and psychological integrity	Case study (Quantitative)	ID specific interventions have a direct impact on justice for complainants with ID who have been sexually abused.
12.	Donohue (2014)	Inclusive education	Opinion and analysis (Qualitative)	Lack of clear policy is the most significant constraint to inclusive education.
13.	Donohue et al. (2014)	Risks to realisation of rights	Case study (Quantitative)	Risk influences access to resources for CWID.
14.	Donohue (2015)	Realising inclusive education	Case study (Quantitative)	Providing teachers with inclusive training could positively influence their attitudes.
15.	Donohue et al. (2015)	Risks to realisation of rights	Case study (Quantitative)	Household size mediated by poverty has communication outcomes for CWID.
16.	Du Plessis (2013)	Realising inclusive education	Opinion and analysis (Qualitative)	South African out-of-school figures for CSPID are consistent with other developing countries in that only 2% of CSPID receive any schooling.
17.	Engelbrecht (2003)	Realising inclusive education	Case study (Mixed method)	Including learners with ID in mainstream classes is stressful for teachers.
18.	Erasmus (2016)	Realising inclusive education	Case study (Quantitative)	Education and safety of most concern to parents.
19.	Geiger (2012)	Right not to be discriminated against	Participatory learning and action approach, and iterative process (Qualitative)	Centre-based carer training urgent in low-income settings.
20.	Hall (2016)	Realising inclusive education	Case study (Qualitative)	Identified actions that mainstream schools can execute to enable the resilience of included adolescents with ID.
21.	Huus (2015)	Realising inclusive education	Case study (Quantitative)	Socio-economic factors affect similar self-raters and proxy raters answers.
22.	Huus (2016)	Realising inclusive education	Case study (Quantitative)	Urban caregivers more aware of various rights than rural counterparts.
23.	Kock (2012)	Right not to be discriminated against	Case study (Quantitative)	Adults with ID in SA are stigmatised.
24.	Kramers-Olen (2016)	Right to psychological and bodily integrity	Literature review (Qualitative)	Highlights tension between protecting PWID from exploitation, and the promotion of sexual autonomy. Competency to consent to sexual acts remains an issue.
25.	Kruger (2015)	Realising inclusive education	Opinion and analysis (Qualitative)	Outcome of substantive equality can be undermined by court’s consideration of reasonableness. South African education policy is insufficient to effect substantive equality.
26.	McKenzie (2016)	Right not to be discriminated against	Case study (Qualitative)	PWID require care; protection from crime, violence and abuse; help with independence, accessing resources, and community integration.
27.	McKenzie et al. (2016)	Risks to realisation of rights	Case study (Qualitative)	Improved support services appropriate to the resources in LAMIC are needed especially when existing FCGs are no longer able to provide care.
28.	McKenzie (2013)	Participation	Grounded theory study (Qualitative)	The Interactive Discourse appears to be related to dynamic, environmental conceptions of disability where competence is built through social interaction.
29.	McKenzie et al. (2013b)	Right to access services and be accommodated	Case study (Quantitative)	Facilities have limited access to general health care. Little emphasis placed on competence and quality of life. Employment and vocational training is neglected. Access to support is limited but emerging.
30.	McKenzie et al. (2013c)	Right to access services and be accommodated	Case study (Quantitative)	Pay closer attention to awareness raising (Article 8, UNCRPD) for community integration. Forms of participation per Articles 29 and 30 were not evident in the study.
31.	McKenzie (2012a)	The right to competence and capacity	Grounded theory study (Qualitative)	The three rights claims (participation, special services and protection) should be re-examined through the lens of an ethics of care that enables participation, and reconsiders reciprocity and interdependence. Medico-psychological gaze regulates educational experience of CWID.
32.	McKenzie (2012b)	Realising inclusive education	Grounded theory study (Qualitative)	Medico-psychological gaze maintains unilateral disability expertise and inherent power relations, but PWID are not one-sided recipients of care and special services.
33.	Nash (1992)	The right to competence and capacity	Opinion and analysis (Qualitative)	Main issues involved in sterilisation include a valid assessment of the level of ID, availability of alternative means of fertility control, and complex ethical factors.
34.	Nel (2007)	The right to competence and capacity	Action research (Qualitative)	Emphasising the coaching modality, a single-unit longitudinal approach ensured successful transition.
35.	Nel (2011)	Realising inclusive education	Action research (Qualitative)	Differential learning can realise the right to life.
36.	Ngwena (2012)	Realising inclusive education	Opinion and analysis (Qualitative)	Differences in embodied ability should be understood in a non-hierarchical and non-separatist manner. Equality jurisprudence must rethink the meaning of difference in order to guarantee inclusive citizenship.
37.	Ngwena (2013)	Realising inclusive education	Opinion and analysis (Qualitative)	State ambivalence towards inclusive education is demonstrated.
38.	Petersen (2004)	Right to access services and be accommodated	Case study (Quantitative)	Demand for ID services are great.
39.	Phasha (2009)	Bodily and psychological integrity	Grounded theory study (Qualitative)	Professionals impact on under reporting abuse and opportunities for prevention.
40.	Phasha (2012)	The right to competence and capacity	Phenomenological study (Qualitative)	Practices that reinforce school-based sexual violence are identified.
41.	Phasha (2013)	Bodily and psychological integrity	Grounded theory study (Qualitative)	Under reporting is influenced by communication barriers, family-related factors, and lack of expertise of the part of professionals.
42.	Phasha (2014)	Bodily and psychological integrity	Phenomenological study (Qualitative)	Vulnerability factors suggest that efforts to mitigate problem of sexual abuse of PWID should occur at individual, family, and community level.
43.	Phaswana (2013)	The right to competence and capacity	Observational study (Case Control) (Quantitative)	Victims with severe ID were more often assessed as unable to testify. Ability to testify should depend on individual factors.
44.	Pillay (2000)	Bodily and psychological integrity	Case study (Qualitative)	Many individuals with ID understand, and are able to relate in court, what happened to them.
45.	Pillay (2003)	The right to competence and capacity	Observational study (Cross-sectional) (Quantitative)	Rural CWID have higher levels of social maturity than urban CWID, perhaps indicative of environmental challenges and development of social competence.
46.	Pillay (2008)	The right to competence and capacity	Case study (Quantitative)	Over 90% of PWID were able to testify, although 60% were not able to make an informed decision to consent to sexual intercourse. Distinctions in competency must be made irrespective of IQ-level.
47.	Pillay (2010)	The right to competence and capacity	Case study (Quantitative)	Majority of sexual assault survivors with ID believe perpetrators should be imprisoned – indicates need for retribution and emotional response to hurtful experience.
48.	Pillay (2011)	Right to access services and be accommodated	Case study (Quantitative)	PWID need help with obtaining social grants and school/occupational placements.
49.	Pillay (2012a)	The right to competence and capacity	Opinion and analysis (Qualitative)	Facilitate testimony testimony while minimising discomfort and prejudicial treatment (like forensic testing of victims with ID).
50.	Pillay (2012b)	The right to competence and capacity	Opinion and analysis (Qualitative)	Access to justice should be easier, not more difficult, for PWID.
51.	Pillay et al. (2008)	The right to competence and capacity	Opinion and analysis (Qualitative)	Forensic mental health practice should better serve the needs and rights of rape survivors with ID.
52.	Roberts (2016)	Risks to realisation of rights	Observational study (Cross-sectional) (Quantitative)	Unmet dental needs of CWID is high.
53.	Rohleder (2009)	Protection against abuse	Case study (Qualitative)	Tension exists between human rights discourse and of restriction of sexual behaviours.
54.	Saloojee (2007)	Right to access services and be accommodated	Case study (Quantitative)	CWID more excluded from services than physically disabled children.
55.	Shabalala (2011)	Bodily and psychological integrity	Observational study (Case Control) (Quantitative)	Higher rates of PTSD and symptom intensity in PWID and history of sexual abuse. Nonsexual traumatic events also result in psychological distress for PWID.
56.	Slone (1998)	Availability of appropriate services	Case study (Quantitative)	Referral of individuals with Mild ID was low from low-SES areas compared to high-SES areas; complicated by inadequate referral structures.
57.	Slone (1999)	Availability of appropriate services	Case study (Quantitative)	Comorbidity not higher than previously reported; higher dual diagnosis prevalence in high-SES areas. No gender differences found. Study incorrectly reports support for positive relation between severity of ID and pervasiveness of psychopathology (correlation is negative).
58.	Spangenberg (2016)	Realising inclusive education	Case study (Qualitative)	CSPID database will have multiple benefits for inclusion and services.
59.	Van Niekerk (2015)	The right to competence and capacity	Observational study (Cohort) (Quantitative)	SE services can be considered as a viable option in resource-constrained environments. Providers of SE services will need to modify approaches in order to meet contextual realities.

ID, intellectual disability; PWID, people with intellectual disability; CWID, children with intellectual disability; CSPID, children with severe and profound intellectual disability; PTSD, post-traumatic stress disorder; UNCRPD, United Nations Convention on the Rights of Persons with Disabilities; LAMIC, low- to middle-income countries; FCG, family caregivers; SES, socioeconomic status; SE, supported employment.

Individual study findings were synthesised into 10 sub-themes. These were then integrated into four main themes (Table 5). The first main theme speaks to the right not to be discriminated against (*n* = 36) and addresses inclusive education, inclusive communication and the right to be understood, acknowledgement of competence and capacity to be included, and socio-political participation and inclusive citizenship. The second theme addresses the right to psychological and bodily integrity (*n* = 11) in terms of protection against abuse, and the right of abuse victims to appropriate treatment. Thirdly, the right to be accommodated (*n* = 8) speaks to availability of appropriate services and subsequent access to these services. Finally, main theme four touches on challenges to rights realisation (*n* = 4), warns of barriers to support and considers obstacles to rights implementation.

**TABLE 5:** Themes and sub-themes.

Themes	Sub-themes
Right not to be discriminated against (*n* = 36)	Inclusive education
	Inclusive communication (the right to be understood)
	Acknowledgement of competence and capacity to be included
	Socio-political participation (inclusive citizenship)
Right to psychological and bodily integrity (*n* = 11)	Protection against abuse
	Right of victims of abuse to appropriate treatment
Right to be accommodated (*n* = 8)	Availability of appropriate services
	Access to available services
Challenges to realisation of rights (*n* = 4)	Barriers to necessary levels and kinds of support
	Obstacles to rights implementation

The majority of included studies pertain to the right not to be discriminated against (*n* = 36) with inclusive education (*n* = 14) and acknowledgement of competence and capacity (*n* = 13) also enjoying focus. The right to psychological and bodily integrity (*n* = 11) enjoyed slightly more research interest than the right to be accommodated (*n* = 8) and challenges to realisation of rights (*n* = 4).

Rights issues that received the least amount of research attention throughout the review period include the right to be understood (*n* = 4) and availability of appropriate services (*n* = 2). Results show that the right to socio-political participation and inclusive citizenship requires urgent investigation (*n* = 1).

The Bill of Rights (Act 108 of 1996) enshrines universal dignity (s7.1), equality (s9.2), protection against discrimination by persons or the state on grounds of disability (s9.3), life (s11), bodily and psychological integrity (s12.2), voting in elections (s19.3a), health care (s27.1a) and primary and secondary school education (s29.1a) (Republic of South Africa [RSA] 1996). Although the Constitution and Bill of Rights still guides South Africa’s post-democratic growth, ID remains excluded from socio-political discourses on equity and transformation. If meaningful transformation is only reached by including the whole of society, excluding PWID risks further discrimination and rights abuses of millions of South Africans.

### Right to not be discriminated against

The right to inclusive education, inclusive communication, competence and capacity, and socio-political participation speaks to issues of discrimination (*n* = 36).

Popular belief that PWID are inferior opens them up to abuse and exploitation (Phasha & Myaka 2014). As vulnerable targets of stigmatisation and disenfranchisement, such discrimination is hard to overcome without support and advocacy (Carey 2003; Kamga 2016). Younger adults with ID from lower socio-economic areas who also have physical disabilities experience additional stigma (Ali et al. 2015). Disability exacerbates complicated issues of race and identity in South Africa, and black individuals with mild ID experience more stigma than white and mixed race PWID (Ali et al. 2015).

#### Inclusive education

The Bill of Rights entitles children *and* adults to basic education (s29.1a, RSA 1996). South Africa also interpreted and used international declarations to transition towards greater inclusion in education. The education system was meant to adjust in ways that would accommodate diverse learners’ needs as inclusively as possible (Engelbrecht et al. 2003).

After the Western Cape High Court^3^ was petitioned on claims to education in 2011, the state was ordered to ensure – within reason – affordable access to adequate education for children with severe and profound ID (CSPID) (Du Plessis 2013; Mckenzie et al. 2017). The White Paper on the Rights of Persons with Disabilities (2016) now reinforces the enrolment of children with any severity of ID previously refused access (DSD 2016). Although the right to basic education is immediate, the state can still exclude PWID from educational settings by arguing reasonable grounds (Kruger 2015; Ngwena 2013). Up to 260 000 school-going aged children with disabilities in South Africa were not enrolled in 2001 – by 2009, the figure amounted to 467 005 (Du Plessis 2013).

The state discriminates against CSPID, in particular, by using severity of impairment to disqualify them from compulsory education (Ngwena 2013). Most enrolled children with mild and moderate ID are separated into schools for learners with special educational needs (LSEN), whereas CSPID are further segregated in special care centres (Donohue & Bornman 2014; Spangenberg et al. 2016). By such segregation, educational policy and schooling for CSPID has become a site of disablement and degradation (Geiger 2012; Human Rights Watch 2015; Ngwena & Pretorius 2012).

Few urban CSPID under 4 years attend special care centres (Spangenberg et al. 2016), and even fewer do in rural areas where prevalence is higher but care centres in shorter supply (Huus et al. 2016). Although rural and urban caregivers are equally aware of rights to education (*p* = 0.001), urban caregivers believe more firmly that CWID have rights (*p* < 0.001) (Huus et al. 2016).

Including PWID in non-discriminatory educational communities is complicated. People with intellectual disability have vastly diverse educational needs and meeting them equitably will require the entire South African mainstream school-LSEN-care centre split system to change (Engelbrecht et al. 2003; Erasmus et al. 2016; Hall & Theron 2016). Adapting curricula for CWID in regular or mainstream classes will demand effort and perhaps impact negatively on educators’ views of inclusion, but training and support can facilitate inclusive attitudes (Donohue & Bornman 2015; Engelbrecht et al. 2003; Hall & Theron 2016; Mckenzie & Macleod 2012b). Addressing diverse educational needs can lead to flexible teaching of CWID in regular schools, accessible assessment practices and learning life preserving skills (Mckenzie & Macleod 2012b; Nel, Kempen & Ruscheinski 2011; Rohleder & Swartz 2009).

#### Inclusive communication

Augmentative and alternative communication (AAC) can support PWID who have difficulties with conventional communication in various settings (Bornman & Alant 1996, 2002; Bornman et al. 2011; Dada et al. 2013). Making little effort to understand what PWID who have communication difficulties are saying, despite the availability of AAC, hinders the right to report rights violations (Pillay 2012a). Enhancing reciprocal communication – realising the right to be understood – can raise self-advocacy opinions and reduce continuous victimisation (Bornman et al. 2011).

#### Competence and capacity

Where competence relates to the ability to do something successfully or efficiently, capacity speaks to legal independence and participation during legal proceedings (UN 2006). Having one’s experiences and accounts of events received in a court of law, for example, is important for building competence (Bornman et al. 2011; Mckenzie 2013; Nash & Navias 1992).

**Competence:** Environmental challenges early in life may inadvertently contribute to developing protective social maturity and competence in CWID (Pillay 2003). Such competence might assist those leaving school with transitioning into gainful work placements aimed at further skills development (Nel, Van der Westhuyzen & Uys 2007; Van Niekerk et al. 2015).

Female learners subjected to school-based sexual violence defy misconceptions that PWID lack comprehension that their rights are violated, or lack competence to respond accordingly (Phasha & Nyokangi 2012). Disclosures of coercion, and of being threatened and humiliated for refusing male learners’ sexual advances, evince awareness of rights to bodily and psychological integrity (Phasha & Nyokangi 2012).

Focusing only on biomedical limitations can overshadow individual competencies, lower expectations for new learning, minimise subjective experiences of living with ID and reinforce unequal power relations (Donohue & Bornman 2014; Geiger 2012; Mckenzie & Macleod 2012a; Young & Berry 2016).

**Capacity:** Denying capacity legitimises discrimination and legally reinforces social prejudice against PWID (Pillay 2012a; UN 2006). Relative to severity level (e.g., mild, moderate, severe or profound ID), adults with mild and moderate ID can negotiate consensual sexual relationships with necessary support and guidance (Hough 2012; Phasha & Miyaka 2014; Reinders 2008). Yet in cases of sexual violence perpetrated against PWID, the burden to prove competency that consent was refused is on the survivor with ID (Pillay 2008, 2012a, 2012b). Despite the survivor’s ability to give an account, legal determination of capacity to testify against the perpetrator is ultimately out of the survivor’s hands and depends on results of mental health examinations (Phaswana, Van der Westhuizen & Krüger 2013; Pillay 2008, 2010, 2012a; Pillay & Kritzinger 2008; Pillay & Sargent 2000). Augmentative and alternative communication can enable courts to consider various forms of non-conventional proof of capacity and competence to give testimony (Bornman et al. 2011).

#### Inclusive citizenship

Citizenship provides legal status and involves participating freely in a particular national space on condition of honouring various rights and duties (Yeung, Passmore & Packer 2008). When viewed as a politically ‘unfit’ homogenous group, the political rights of people with intellectual impairment are disabled in cases where individuals have competence and capacity to make political decisions (Dowse 2009; McDonagh 2008; McKenzie & Adnams 2014; Smith, Foley & Chaney 2008; Stein & Stein 2007).

The medico-psychological model’s prescription of special health services and protections, though warranted, risks the independence of PWID to apply their political and participatory rights as citizens with political choices (McKenzie & MacLeod 2012b). However, equitable participation (voting), appropriate services (assistive technology) and protections (against voter intimidation) can dispel the assumption that specialist services, protections and supports are impossible to reconcile in order to realise the political rights of PWID (McKenzie & MacLeod 2012b; Pieterse 2006).

The UNCRPD’s Article 8 addresses prejudice and stereotyping, and Articles 29 and 30 pertain to political and social community participation (UN 2007). Yet, safe inclusion of PWID as members of South African communities is yet to be aligned with these principles (McKenzie, McConkey & Adnams 2013b).

### Right to psychological and bodily integrity

The second theme addresses the right to psychological and bodily integrity (*n* = 11).

Despite attempts at advancing legislation and public awareness around ID, individuals with ID appear to be easy targets for perpetrators of sexual violence. Sexual abuse of teenagers with ID is widespread, yet they experience compounded difficulties when navigating the justice system. In light of the vulnerability to sexual assault and becoming a victim of crime in South Africa, communicative capacity and being understood becomes especially important during subsequent legal proceedings

#### Protection against abuse

Female PWID are at increased risk of sexual abuse, that of teenagers with ID is common, and close relatives are implicated in most cases of sexual assaults against CWID (Bornman & Rathbone 2016; Calitz et al. 2014; Meel 2009; Phasha 2009, 2013). Perpetuating such rights violations are misconceptions that PWID have a high sex drive, possess unusual power and feel no pain, and that sex with PWID is an act of pity and can cure the perpetrator of disease (Phasha & Myaka 2014).

Survivors with ID are particularly vulnerable to psychological effects of sexual assault and present with higher rates of post-traumatic stress disorder and symptom intensity than ID individuals with non-sexual traumatic histories (Shabalala & Jasson 2011). Given these increased vulnerabilities, it is unthinkable to exclude PWID from sexual education programmes (Yildiz & Cavkaytar 2017). Without access to sexual health education, PWID will continuously struggle to protect themselves against unwanted sex, pregnancy and transmittable infections (Bornman & Rathbone 2016; Meel 2009; Rohleder & Swartz 2009).

Dispelling harmful misconceptions about ID and sexuality might initiate services, policies and beliefs that are more supportive of significant relationships. People with mild and moderate ID can develop meaningful sexual relationships by learning about, and consenting to and maintaining healthy sexual behaviours (Bornman & Rathbone 2016; Kramers-Olen 2016; Phasha & Myaka 2014; Rohleder & Swartz 2009). Individuals with severe and profound ID will find it harder to negotiate consent around sexual practices. Interventions aimed at reducing incidence of sexual abuse of PWID can occur in formal educational and informal community settings (Phasha & Myaka 2014). For example, traditional healers hold high esteem among many South Africans, and customary notions that jeopardise rights of PWID can be reconceptualised with these individuals (Kromberg et al. 2008; Phasha & Myaka 2014).

#### Right to appropriate treatment: Health, justice and well-being

Tackling ID needs at primary health care level can advance universal services and foster integration of PWID into the national health system (Molteno, Adnams & Njenga 2011; Petersen 2004; Pillay & Siyothula 2011). High rates of sexual violence warrant mental and physical health interventions for PWID at primary health clinics (Phasha 2013; Shabalala & Jasson 2011).

We appreciate that sexual assault cases involving complainants with ID challenge the South African investigative and judicial systems (Calitz 2011; Pillay & Kritzinger 2008). Decisions about an ID survivor’s ability to testify should not only depend on measures of intellectual functioning but consider self-determination as well (Phaswana et al. 2013). Appropriate mental health and legal services for ID rape survivors can aid justice and equality under the law (ss9.1 and 9.2) (RSA 1996).

The Sexual Abuse Victim Empowerment (SAVE) programme exemplifies a not-for-profit project established for complainants with ID in sexual assault cases (Dickman & Roux 2005). Since 1990, SAVE has advised investigators and prosecutors, addressed complaint-specific service needs like court preparation and availed expert testimony (Dickman & Roux 2005). By 2005, conviction rates similar to the best rate for sexual assault cases in the general population were achieved (Dickman & Roux 2005). Since then, justice for ID victims of sexual assault has lost some ground (Pillay 2012b), and the White Paper on the Rights of Persons with Disabilities admits that police lack disability service skills and have reservations about responding to complaints on behalf of PWID (Department of Social Development 2016).

### Right to be accommodated

The lack of reliable and affordable public transport infrastructure and expenses incurred to access private transport, as well as the high rate of human immunodeficiency virus infection and acquired immune deficiency syndrome (HIV and AIDS) and its prioritisation in the primary health care system, serve to complicate access to state-subsidised primary care medical services throughout South Africa. The right to be accommodated (*n* = 8) speaks to subsequent access to available and appropriate services for PWID.

#### Availability of appropriate services

Most PWID in South Africa likely suffer poor nutrition and live in socio-economically distressed areas that further predispose them to negative outcomes (Pillay & Siyothula 2011; Slone et al. 1998, 1999). The majority of families who care for dependent PWID rely on monthly social grants of R1600 (± USD124 at the time of writing) (South African Social Security Agency 2017), and one in four families frequently go without food before their next grant pay-out (Pillay & Siyothula 2011).

Unmet service needs of PWID combined with poverty create a dire situation for PWID (Adnams 2010; De Vries et al. 2013; McKenzie et al. 2013b; Saloojee et al. 2007; Tomlinson et al. 2014). Nonetheless, there is little evidence of service integration among South Africa’s health, education and social development departments in meeting these needs (Saloojee et al. 2007), whereas the opportunity costs of informal ID care remain unrecognised (McKenzie et al. 2013b).

Further, rights concerns pertain to ill-treatment of PWID by health workers (Newton & McGillivray 2017). Disconcertingly, primary health nurses are potential sources of support and often the first professional contact for families caring for PWID (Bornman & Alant 2002). Ill-treatment of PWID and their caregivers could be reduced by up-skilling primary health care professionals with knowledge of ID care (Bornman & Alant 2002). Community-based services for PWID can be supported by implementing and monitoring task-shifting of appropriate assessment, intervention and referral services (Geiger 2012; Petersen 2004; Shabalala & Jasson 2011).

#### Access to available services

People with intellectual disability in South Africa remain excluded from services that could encourage their well-being (Adnams 2010; McKenzie, McConkey & Adnams 2013c; Molteno et al. 2011). Children with intellectual disability, for example, are five times less likely to receive rehabilitation services than physically disabled children (*p* < 0.0001) (Saloojee et al. 2007).

Owing to funding difficulties, community homes for adults with ID focus on custodial rather than participatory socio-political practices (McKenzie et al. 2013c). Although community-based residential and occupational programmes can enable socio-economic participation (DSD 2016), the misconception that PWID are economically unviable or unable to contribute financially to their communities violates their socio-economic rights (Dowse 2009; McDonaugh 2008). Adults who contribute in the form of child care, household chores and social grants feel exploited rather than valued for their role (McKenzie 2016; McKenzie et al. 2013c).

Adults with ID who also have behavioural, physical and mental health difficulties face multiple barriers to accessing necessary services (De Vries et al. 2013; McKenzie et al. 2013b). Mentally ill PWID may experience marginalisation in an amplified manner by being sequestered as less interesting yet more bothersome members of disabling societies. Given the direction of power in such settings, any protest on their part or ‘suggestions … about care are likely to be taken as resistance or obstruction’ (Tronto 2010:165), confirming their status as less than equal adults.

### Challenges to rights realisation

Finally, challenges to realisation of rights (*n* = 4) warns of barriers to necessary levels and kinds of support and considers obstacles to rights implementation. People with intellectual disability struggle to exercise their constitutional rights when confronted with service barriers and low political prioritisation of care (Department of Health [DoH] n.d.; Donohue, Bornman & Granlund 2014; Huus et al. 2015). Rights to health are hampered by shortages of professionals with ID training, unavailable medications typically indicated for treatment, obstructive referral pathways, travelling to multiple medical facilities for various interventions and poverty (Huus et al. 2015).

The majority of children with disabilities in South Africa do not attend compulsory school and a lack of clear policy constrains inclusive education (Donohue & Bornman 2014, 2015). Children with physical disabilities who also have ID will most likely be excluded from receiving assistive devices and rehabilitation (Alper & Goggin 2017; Boot et al. 2017; Donohue et al. 2014, 2015; Saloojee et al. 2007; Spangenberg et al. 2016). Moreover, CWID are at increased risk of abandonment, abuse, multiple handicap, behaviours that challenge but may be phenotypical, co-morbid psychiatric difficulties, preventable illnesses and poor physical and dental health (De Vries et al. 2013; Dickman & Roux 2005; Giarelli et al. 2009; Molteno et al 2001; Pillay 2012a, 2012b; Pillay & Kritzinger 2008; Roberts et al. 2016; Van Rensburg 2007).

Most adults with ID in South Africa are cared for by family and predominantly so by women. Caregiver experiences of isolation and minimal support are common and similar across race and class (McKenzie 2016; McKenzie & McConkey 2016). Care burdens that restrict personal growth and opportunities to pursue employment contribute to caregiver stress and pose risks to sustainable care resources (Coetzee 2015; McKenzie & McConkey 2016).

## Discussion

Underestimating pervasive ableism in South Africa trivialises the exclusion of PWID from realising their rights. Public infantilisation, abuse and taunting perpetuate the isolation of PWID who may find themselves caught between negative public perceptions and attempts at community, occupational and socio-political inclusion. South Africa’s political rights (s19.3a, RSA 1996) indiscriminately entitle prison inmates to vote in elections, whereas citizens with ID face multiple barriers to exercise this right (Combrinck 2014; Hartley 2013; Kopel 2017; Ndenze 2013; Swart 2015). South Africa’s ‘unsound mind’ aphorism maintains an outdated justification for disenfranchising PWID regardless of contemporary support (Article 29a, UN 2006) and successful suffrage elsewhere in the world (Hood 2014; Kjellberg & Hemmingsson 2013; The Electoral Commission UK 2015).

Denial of competence and legal capacity are also fundamental ID rights violations that perpetuate discrimination and exclusion. Given contemporary interpretations of legal capacity and equality under the law (Article 12, UN 2006), the onus of proving that PWID are (un)able to participate in legal proceedings that impact their lives should be on the court – not on the survivor with ID.

People with intellectual disability are often targets of sexual violence, but obtaining justice for survivors with ID is difficult in South Africa. We thus question whether sexual assault survivors who have ID, after suffering trauma and perhaps undergoing a medico-legal examination, must undergo still further evaluations before being deemed fit to testify to their account of events (Pillay 2008). Why should rape survivors with ID have to submit to testing at all, as opposed to perpetrators proving they were unaware of their victims’ intellectual impairment? Having to competently ‘pass’ a mental health exam prior to having capacity bestowed makes it harder for PWID to obtain justice, not easier. This approach to litigation might actually violate the dignity, equality, bodily and psychological integrity, and protection of PWID against discrimination by the state on grounds of disability (RSA 1996). Having ratified the UNCRPD (UN 2007), should the state not honour Article 12.3 and provide any means necessary to equitably meet the needs of PWID in the criminal justice system? The forensic examination of sexual assault survivors with ID should be critically (re)considered – no matter the levels of support required in the absence of mental health testing (Prinsloo 2008; Tronto 2010).

Furthermore, justice for offenders with ID must also be considered. Offenders may understand truth, lies and moral wrongfulness but be less able to link these to real implications. Knowing right from wrong should be differentiated from successful applications of social insight and adaptive skills in appreciation of the consequences of wrongfulness. We question denying ID defendants the opportunity to testify in their own defence. A finding of intellectual impairment need not summarily disqualify one from testifying, because defendants with comparatively better adaptive than intellectual functioning can be triable (Calitz et al. 2007; Pillay 2012a, 2012b). The disenfranchising South African ‘unsound mind’ aphorism is again criticised, because PWID are not automatically without capacity (Combrinck 2014; Phaswana et al. 2013).

In returning to our ID definitions, legal capacity requirements that emphasise adaptive abilities might challenge a medical underscoring of intellectual deficit. Because abilities to observe, remember and communicate can be established during testimony, defendants might be enabled to give evidence – if so advised by legal representation – notwithstanding conventional ‘test’ findings of intellectual deficit (Pillay 2008, 2012a, 2012b; Pillay & Kritzinger 2008).

Thus far, we have commented on the vulnerability of PWID who are victims and perpetrators of crimes. But what of any adults with ID in South Africa? By interpreting the United Nations Convention on the Rights of the Child (UNCRC) (UN 1989), the protection of vulnerable children is legislated in South Africa’s *Children’s Act* (38 of 2005, Department of Justice [DoJ] 2005). Consequently, the departments of Social Development and Health are legally required to intervene on behalf of children at risk. The *Mental Healthcare Act* (DoH Act 17 of 2002) and General Regulation Amendment (DoH 2016) supports institutional state care for adults with severe and profound ID, but lacks rights governance for adults with any severity of ID living in community settings. Beyond the inadequate *Mental Healthcare Act* (DoH 2002) or even the *Older Persons Act 13* (DoJ 2006), no act similar to the *Children’s Act* (DoJ 2005) intervenes on behalf of vulnerable adults with ID at risk of rights abuses and death (Makgoba 2016).

In light of the lacking legislation as discussed above, we turn to the Esidimeni care crisis, violations of the constitutional right to life (RSA 1996) and the urgency of purposeful community safety requirements of PWID. The Esidimeni crisis started in 2016 and marks the deaths of more than 140 adults in community care after being discharged from a specialist care facility by the Gauteng Health Department (Bornman 2017; Lund 2016; Rahlaga 2017; Tlhabye 2017). Although at least half of the deceased lived with ID in the absence of psychiatric illness, a well-intended Ombud report (Makgoba 2016) obscured distinctions between mental illness and ID and thus dismissed the particular vulnerabilities and service needs of individuals who live with either or both. With no statutory framework or legislated minimum threshold of community care to answer to yet, the state currently maintains no legal obligation to perform adequate safeguarding of adults with ID.

Despite deinstitutionalisation, PWID remain isolated from communities in which they feel victimised, rarely participate in basic education and skills development programmes and struggle to find appropriate work placements without help. The need remains for protective and participatory community-based services, but these must be developed and monitored in line with purposeful care and safe community inclusion. This is particularly urgent as results have shown. When family caregivers burn out or die, PWID are left with limited options. Few relatives or neighbours willingly take on the support needs of PWID (Geiger 2012), whereas community care facilities are in short supply and have years-long waiting lists.

As it is the right to inclusion of every person in South Africa, PWID should also be able to have their health needs initially assessed or met at primary health care level, yet are often redirected to specialist settings for services that could have been delivered at their local clinics. In the same vein, a lack of ID awareness at primary care level results in PWID getting lost to referral services in cases where they should have been receiving specialist intervention.

We end this discussion by suggesting that one way to reduce discrimination against PWID is to encourage equitable practices that include and rely on the expertise of people living with ID. In just societies that celebrate self-determination, assistance is commonplace, and barriers to developing adaptive abilities are removed to increase competency.

## Practical implications

In returning to our aims and objectives, we now see that PWID in South Africa face more difficulties than the general population when attempting to access justice, health, educational, employment and social services. Prejudice increases violence against PWID, but negative bias excludes them from public health and safety campaigns. There is a need to address such discrimination through advocacy interventions similar to those employed in other national rights campaigns, like the HIV/AIDS Treatment Action Campaign^4^ (tac.org.za).

Legislation for vulnerable adults with ID is yet to be advocated for and taken up in protections, services and policies in South Africa. An adult at risk is any person who is aged 18 years or over and at risk of abuse or neglect because of their needs for care or support. We can cautiously turn to just as recent European examples of *the United Kingdom Care Act* (DoH UK 2014), Safeguarding Policy of the Office of the Public Guardian (Office of the Public Guardian England and Wales 2015), Vulnerable Adults Act Draft Bill (Government of Singapore 2016), *Protection of Vulnerable Adults from Financial Exploitation Act* (Alabama Securities Commission 2016) and the *Adult Protective Services Act* (State of Illinois Department on Aging 2013). These examples share the common goal of preventing harm and reducing the risk of abuse or neglect to adults with care and support needs, and safeguarding adults in ways that support them in making choices and having control in how they choose to live their lives. In South Africa, such legislation can raise public awareness so that individuals and communities can play their part in preventing, identifying and responding to the abuse and neglect of vulnerable adults living with ID.

We all require opportunities to realise our constitutional rights. People with intellectual disability require encouragement to participate in the socio-political lives of their communities, whatever forms these take. Sufficient support will exceed minimal levels but must be provided – whether these offers are taken up will remain every individual’s choice, but must be made available (Stein & Stein 2007; Tronto 2010). Exercising ID rights implies negotiating *not only* with individuals who aim to support ID voices, dismantle restrictions and develop political behaviours *but also* with those who prefer to maintain barriers. Because compliance and resistance to inclusive processes can be expected, self-advocacy groups and local ID organisations must be sustained.

Students from various disciplines enthusiastic about working with PWID will be better prepared for meeting ID care needs if trained as integrated practitioners (Geiger 2012; Roberts et al. 2016). Instructors with ID can broaden practitioner understanding, and future service designs can integrate the experience of PWID and their caregivers (Grut et al. 2012). Continuous professional development and journal clubs can keep practitioners current on best practice (Donohue & Bornman 2015).

Rights realisations for PWID requires eliminating stigma, encouraging inclusive practices, opportunities for occupational skills development, access to job coaching and supportive employers and pathways out of poverty. Dispelling stereotypes and presumptions, campaigning for quality primary ID health care, advocating for legislative support and lobbying political will could reduce the risk of life-threatening discrimination against PWID (Makgoba 2016).

## Implications for research

South African ID rights researchers have aligned their arguments with the Bill of Rights (RSA 1996) and global disability initiatives like the UNCRPD (Drew et al. 2011; UN 2006). Knowledge on ID is still predominantly located in non-intellectually impaired individuals, mostly at universities from which PWID are excluded. If projects are not driven by people with intellectual impairment, their experiences of disablement must at least be included during service development and research planning phases.

Although inclusive research locates PWID as co-researchers, emancipatory research encourages principle researchers with ID to select topics, collect and analyse data and publish in accessible journals with necessary support as required. Both approaches can realise the right of PWID to create and claim knowledge on ID matters (Capri & Coetzee 2012). Consulting with self-advocates and researchers with ID can not only change the way ID is understood and responded to in South Africa but also collect opinions of PWID on issues that affect their lives directly.

Rights issues that received the least amount of research attention throughout the 25-year review period include the right to be understood (*n* = 4) and the lack of appropriate services (*n* = 2). The right to socio-political participation and inclusive citizenship requires urgent investigation (*n* = 1). There are opportunities for researching the experiences and attitudes of primary health care workers towards PWID and for addressing a lack of South African evidence regarding support and interventions for offenders with ID. The sexual rights and health of PWID, and rights to parent, can be included as neglected fields of study and advocacy.

## Limitations

We aimed to describe published studies on advocacy, addressing stigma and promoting ID rights and awareness in South Africa. It is beyond the scope of this review to include work on ID in South Africa that has not been published in peer-reviewed journals (i.e. ‘grey literature’), and this limitation can contribute to publication bias.

Although arguments on therapeutic sterilisation were rationally and logically presented by Nash and Navias (1992) over two decades ago, it must be noted that the context of sterilisation of PWID in South Africa has changed significantly since 1992. The authors uncritically argue that persons with mild ID would not be able to provide consent for sterilisation. The findings of this particular article might have been acceptable at the time, but are certainly not tolerable in 2017 (at the time of writing), and it is doubtful whether it would garner support from current clinicians in the field.

Donohue et al. (2014) recognise that their results are limited by the sample of CWID selected to participate in their study and that their findings cannot be generalised as representative of experiences of all CWID in South Africa. Despite inclusive education service delivery problems, we must highlight that there are approximately 423 schools for LSENs in South Africa.

## Conclusion

Our scoping review investigated pertinent issues relating to the rights of PWID in South Africa. We incorporated available research evidence in a synopsis of 59 eligible studies, addressed implications for practice and identified areas for future investigation. Realising the rights of PWID in South Africa to participate as socio-political equals, access services, own their psychological and bodily integrity, and move freely without discrimination is an ongoing project. Barriers to exercising these rights were highlighted. Research evidence advocates that – for a start – the rights and needs of PWID be taken up with serious commitment by the South African state, its legislature and public service departments. Statutory obligations to protect and realise the rights of any South African must extend to PWID and their supporters who are forging ahead in a disabling and service constrained socio-political environment.

Turning back to the continuum of ‘changing states of impairment and health’ (Swartz et al. 2012:1), Kittay et al.’s (2005) myth of independence helps us realise that being human can be defined not by commonly shared characteristics (e.g. high-level cognitive functioning), but by what all human beings are not. We are not immune to the injurious fragility of bone and brain, we are not of able mind and body permanence and we are ultimately never independent.

Although still occupying a space on the fairly healthy end of Swartz et al.’s (2011) continuum, we may find it difficult to imagine ourselves in a future impaired, ill or injured state. But we must take cognisance of a universal human frailty so that we can face our own inescapable fragility with dignity and justice, secure in the protection against violence of moral, psychological and physical abuse.

## Footnotes

1 The estimated South African population is 55 908 865 (World Bank 2017), implying that more than 2 000 000 individuals may be living with difficulties ‘in remembering or concentrating’.

2 MeSH (Medical Subject Headings) is the National Library of Medicine (NLM) controlled vocabulary thesaurus used for indexing articles. It facilitates replicability and consists of sets of terms naming descriptors in a hierarchical structure. Articles are associated with a set of MeSH terms that describe the content of the article.

3 Western Cape Forum for Intellectual Disability (WCFID) v Government of the Republic of South Africa and Another (2011) SA 87 (Western Cape High Court, Cape Town).

4 The Treatment Action Campaign (TAC) started campaigning in South Africa for access to AIDS treatment in December 1998. It has since become one of the most impactful civil society organisations working on AIDS in the Global South. By 2002, it successfully campaigned the Constitutional Court to order the South African government to provide anti-retroviral drugs to prevent vertical transmission of HIV from mothers to infants during birth.
